# Mechanical and Physicochemical Properties of Composite Biopolymer Films Based on Carboxymethyl Cellulose from Young Palmyra Palm Fruit Husk and Rice Flour

**DOI:** 10.3390/polym14091872

**Published:** 2022-05-03

**Authors:** Pimonpan Kaewprachu, Chalalai Jaisan, Warinporn Klunklin, Suphat Phongthai, Saroat Rawdkuen, Wirongrong Tongdeesoontorn

**Affiliations:** 1 College of Maritime Studies and Management, Chiang Mai University, Samut Sakhon 74000, Thailand; chalalai.jai@cmu.ac.th; 2 Cluster of Innovative Food and Agro-Industry, Chiang Mai University, Chiang Mai 50100, Thailand; warinporn.k@cmu.ac.th; 3 School of Agro-Industry, Faculty of Agro-Industry, Chiang Mai University, Chiang Mai 50100, Thailand; suphat.phongthai@cmu.ac.th; 4 The Cluster of Agro Bio-Circular-Green Industry (Agro BCG), Chiang Mai University, Chiang Mai 50100, Thailand; 5 Lanna Rice Research Center, Chiang Mai University, Chiang Mai 50200, Thailand; 6 Unit of Innovative Food Packaging and Biomaterials, Mae Fah Luang University, Chiang Rai 57100, Thailand; saroat@mfu.ac.th (S.R.); wirongrong.ton@mfu.ac.th (W.T.); 7 School of Agro-Industry, Mae Fah Luang University, Chiang Rai 57100, Thailand

**Keywords:** biopolymer films, carboxymethyl cellulose, composite films, physicochemical properties, rice flour

## Abstract

Carboxymethyl cellulose from young Palmyra palm fruit husk (CMCy) film has low water barrier properties, which can limit its application. Thus, the combination of CMCy with other polysaccharides, such as rice flour (RF), may solve this problem. The aim of this study is to prepare the CMCy/RF composite films in different proportions (CMCy100, CMCy75/RF25, CMCy50/RF50, CMCy25/RF75, and RF100) and investigate their mechanical and physicochemical properties. The film strength (33.36–12.99 MPa) and flexibility (9.81–3.95%) of the CMCy/RF composite films decreased significantly (*p* < 0.05) with an increase in the RF proportion. Blending the RF with CMCy could improve the water vapor permeability (9.25–6.18 × 10^−8^ g m m^−2^ s^−1^ Pa^−1^) and film solubility (82.70–21.64%) of the CMCy/RF composite films. Furthermore, an increased lightness with a coincidental decreased yellowness of the CMCy/RF composite films was pronounced when the RF proportion increased (*p* < 0.05). However, the addition of RF in different proportions did not influence the film thickness and transparency. Based on SEM micrographs, all film samples had a relatively coarser surface. FTIR spectra showed that some interactions between CMCy and RF blended films had occurred. According to these findings, the CMCy50/RF50 composite film was found to be the best formulation because it has good mechanical and physicochemical properties.

## 1. Introduction

In recent years, many organizations around the world have been actively campaigning to reduce the use of packaging made of non-biodegradable petroleum-based packaging materials, such as plastic. This is because the use of these materials has caused a large amount of waste and they take a long time to degrade, which has had a negative impact on the environment. As a result, biopolymer materials from various renewable resources, such as polysaccharides, proteins, lipids, or combinations of these, have gained worldwide attention for being used as starting materials in biopolymer packaging film production. Currently, biopolymer films are expected to substitute synthetic polymers. This is because biopolymer materials show several advantages over non-biodegradable petroleum-based packaging materials, which include a relative abundance, biocompatibility, biodegradability, film-forming ability, non-toxicity, cost-effectiveness, and recyclability [1,2,3]. These biopolymer films can control the migration of moisture, gas, and lipids, as well as act as carriers for additives and nutrients [4]. Among biopolymer materials, polysaccharides, such as carboxymethyl cellulose and rice flour, have the potential for forming composite films.

Carboxymethyl cellulose (CMC) is a cellulose derivative that is obtained through the carboxymethylation process of cellulose. It is a linear polysaccharide, an anionic, and a water-soluble substance. The water solubility of CMC is due to the carboxymethyl group of the cellulose molecules [5]. It is frequently used in the pharmaceutical, biomedical, cosmetic, plastic, food, and textile industries [5]. The CMC could be synthesized from agricultural by-products that contain cellulose materials. Some include asparagus stalk end [2], durian rind [6], pineapple peel [7], sugarcane bagasse [8], and rice stubble [9]. One type of wood-based plant that can be used for the synthesis of CMC is the Palmyra palm (*Borassus flabellifer* L.) fruit husk, a by-product after removing sweet jelly seeds. In a previous study [1], it was found that the degree of substitution (DS) value of young Palmyra palm fruit husk CMC (CMCy) was 0.51. It had the ability to form films with a light-yellow color, moderate mechanical properties, high water vapor permeability, and relatively low water resistance. Many studies have also reported that CMC film exhibits low water barrier properties, which could limit its application [10]. One technique that is widely used to solve this problem is biopolymer combinations by mixing CMCy with other polysaccharides that exhibit film-forming properties and are obtained from renewable resource materials, such as rice flour.

Rice (*Oryza sativa* L.) is an economically important crop in Thailand. Rice flour (RF) is a starchy material that is obtained from the milling process of broken rice. The main component in RF is carbohydrates, which accounts for around 90% of the dry weight of rice grain, followed by proteins and lipids at 6.5% and 0.8%, respectively [11]. It is a cheap, edible, and abundant material. The starch granules of RF are mainly composed of two polysaccharides: amylose and amylopectin. Amylose plays a major role in forming the film due to its linear structure [11]. Previous research has shown that film made from RF exhibited irregularities in the surface when observed under a surface electron microscope (SEM). It also has low mechanical properties and water solubility [11]. RF can improve the mechanical and physicochemical properties of fish gelatin-based films [12], chicken gelatin-based films [13], and pectin-based films [14]. Thus, blending the CMC with RF can produce film with interesting properties.

The combinations of biopolymer materials to produce composite films with the aim of improving the properties of the resulting composite films have been investigated. Some include CMC/alginate/chitosan [15], cassava starch/CMC [16], rice starch/CMC [17], starch/CMC [18], potato starch/CMC [19], corn and cassava starch/CMC [20], and CMC/chitosan [21]. The mechanical, physicochemical, thermal, and/or gas/moisture barrier properties of the resulting composite biopolymer films might be enhanced based on component interactions within the film structures [22]. The blending of CMCy and RF is expected to form a compatible film with enhanced film properties. However, there is no information about the effect of the combinations of CMCy and RF on the mechanical and physicochemical properties of the resulting composite films. Therefore, the aim of this study was to prepare the CMCy/RF composite films in different amounts (CMCy100, CMCy75/RF25, CMCy50/RF50, CMCy25/RF75, and RF100) and investigate their mechanical and physicochemical properties.

## 2. Materials and Methods

### 2.1. Materials

Young Palmyra palm fruit husk was obtained from Ban Lat district, Phetchaburi province, Thailand. Rice flour (Lieng Tong Rice Flour Co., Ltd., Nakhon Pathom, Thailand) was purchased from a local market in Muang, Samut Sakhon, Thailand. All other chemicals were of analytical grade.

### 2.2. Extraction of Cellulose

Prior to extracting cellulose, the young Palmyra palm fruit husk was washed, cut, and dried in a tray dryer at 70 °C for 24 h. Briefly, 10 g of dried ground sample was mixed with 100 mL of 30% (*w*/*v*) NaOH solution and subjected to heat at 80 °C for 2 h. The residue was bleached with 20% (*v*/*v*) H_2_O_2_ at 70 °C for 3 h, washed with distilled water (DW), and then dried at 55 °C for 24 h. The obtained cellulose was collected and used to synthesize carboxymethyl cellulose [1].

### 2.3. Carboxymethyl Cellulose (CMC) Synthesis

100 mL of water:isopropanol (20:80, *v*/*v*) and 10 mL of 50% (*w*/*v*) NaOH solution were added along with 5 g of the obtained cellulose. The mixture was stirred at room temperature (RT) for 1 h. After that, the carboxymethylation process was synthesized according to the method described by Kaewprachu et al. [1]. The obtained CMC was referred to as “young Palmyra palm fruit husk carboxymethyl cellulose (CMCy)”.

### 2.4. Preparation of Composite Films

To prepare the film-forming solution (FFS), 100 mL of DW was blended with 3 g of CMCy powder and then heated at 80 °C for 10 min [1]. Similarly, 100 mL of DW was mixed with 3 g of rice flour (RF) and heated for 30 min at 85 °C [23]. Each FFS was mixed for 15 min with 30% (*w*/*w*, based on total solid content) glycerol.

The CMCy/RF composite solutions were produced by mixing the above FFS of CMCy and RF in different amounts (CMCy100, CMCy75/RF25, CMCy50/RF50, CMCy25/RF75, and RF100, *w*/*w*). The FFS of blended CMCy/RF was stirred for 5 min and submitted to sonication in a 500 W ultrasonic cleaner (GT sonic-D27, GT Sonic, Meizhou, China) at 55 °C for 15 min, then cast on a silicone plate (50 × 50 mm) and dried in an oven at 30 °C for 24 h. The CMCy/RF was continuously dried in a constant climate chamber (KMF 115, Binder GmbH, Tuttlingen, Germany) at 50 ± 5% relative humidity (RH) and 25 ± 0.5 °C for 24 h. The CMCy/RF composite films were peeled from the silicone plate.

### 2.5. Conditioning of Films

Prior to testing, the CMCy/RF composite films were conditioned for 48 h in a constant climate chamber (KMF 115, Binder GmbH, Tuttlingen, Germany) at 50 ± 5% RH at 25 °C. These composite films were kept in a desiccator for two weeks and used for FTIR and SEM analysis.

### 2.6. Characterization of CMCy/RF Composite Films

#### 2.6.1. Film Thickness

The thickness of the CMCy/RF composite films was determined using a hand-held micrometer (Mitsutoyo Co., Kanagawa, Japan), which had a 0.001 mm precision. Six random positions around each film were measured (10 specimens).

#### 2.6.2. Mechanical Properties

The mechanical properties (tensile strength; TS and elongation at break; EAB) of the CMCy/RF composite films were measured by a Universal Testing Machine (Strograph E-S, Toyo Seiki Seisaku-Sho Ltd., Tokyo, Japan) with a 100 N load cell. Each film (20 × 50 mm) was determined by using a 30 mm grip separation distance at a test speed of 30 mm/min.

#### 2.6.3. Films Appearance and Color

A digital camera (Fujifilm Finepix S4900; acquired from Fujifilm Thailand Co. Ltd., Bangkok, Thailand) was used to photograph the appearance of the CMCy/RF composite films.

The colorimeter (ColorFlex EZ, HunterLab, Virginia, USA) was used for color analysis of the CMCy/RF composite films. Color parameters were expressed as L* (lightness/blackness), a* (redness/greenness), and b* (yellowness/blueness) values.

#### 2.6.4. Film Transparency

The transparency value of the CMCy/RF composite films was taken from the value of light transmittance at 600 nm using a UV-Vis spectrophotometer (Biochrome, Libra S60, B, Cambridge, England) and calculated according to the following Equation (1) [24]:

(1) $$\mathrm{Transparency}=\frac{-\;\log T600}{x}$$

where *T*600 is the fractional transmittance at 600 nm and *x* is the film thickness (mm).

#### 2.6.5. Water Vapor Permeability (WVP)

The water vapor permeability (WVP) of the CMCy/RF composite films was determined using a modified ASTM E96-80 method [25]. The film was sealed onto an aluminum cup (circle of 70 mm in diameter) containing 10 g of silica gel (0% RH). The cup was kept in a constant climate chamber (KMF 115, Binder GmbH, Tuttlingen, Germany) at 75 ± 5% RH at 25 °C for 8 h and weighed hourly. The WVP value of the film was expressed as g m m^−2^ s^−1^ Pa^−1^.

#### 2.6.6. Film Solubility

The CMCy/RF composite biopolymer films (20 × 20 mm) were first dried at 105 °C for 24 h, recording an initial dry weight (*Wi*), and then shaken at 250 rpm for 24 h, containing 10 mL of DW. The undissolved film sample was separated by centrifuging at 3000× *g* for 10 min, then subjected to drying at 105 °C for 24 h, and subsequently weighed (*Wf*). The percentage of solubility of the CMCy/RF composite films was calculated using Equation (2):

(2) $$\mathrm{Film}\;\mathrm{solubility}\;(\%)=\frac{\mathrm{Wi}-\mathrm{Wf}}{\mathrm{Wi}}\;\times \;100$$

where *Wi* and *Wf* are the initial dry weight of the film sample and the final dry weight of the undissolved film sample, respectively.

#### 2.6.7. Fourier Transform Infrared (FTIR) Analysis

The FTIR spectra of the CMCy/RF composite films were analyzed using the Perkin Elmer FTIR Spectrometer Spectrum One (Perkin Elmer, Waltham, MA, USA) operated at a resolution of 4 cm^−1^. The FTIR spectra were recorded in the range of 4000–400 cm^−1^ wavenumber, with 64 scans.

#### 2.6.8. Morphology of Film

The surface morphology of the CMCy/RF composite films was analyzed using a scanning electron microscope (SEM) (JSM-IT500HR, JEOL, Tokyo, Japan). The image was captured using a magnification of 100×, with an acceleration voltage of 10.0 kV.

### 2.7. Statistical Analysis

The data obtained in this study were analyzed with an analysis of variance (ANOVA) procedure using SPSS software (SPSS for Windows version 16.0, SPSS Inc., Chicago, IL, USA). The differences between means were taken using Duncan’s multiple range tests (*p* < 0.05).

## 3. Results and Discussion

### 3.1. Film Thickness

The thickness values of the CMCy film (control film), CMCy/RF composite films, and RF film were 0.056 mm, 0.056–0.057 mm, and 0.058 mm, respectively (Table 1). Among the CMCy/RF composite films, increased levels of RF did not show a significant influence on film thickness (*p* > 0.05). The results suggested that CMCy can be formed into the compactness of the film matrix with RF, as demonstrated by the fact that it did not influence film thickness. According to Tavares et al. [20], the resulting film formed a more compact structure due to the presence of intermolecular interactions between the hydroxyl groups of RF and the carboxyl groups of CMC. The RF film showed the thickest film compared with those films prepared from CMCy and blended CMCy/RF (*p* < 0.05). Ahmad et al. [12] found that the RF film had the highest film thickness (0.041 mm) and the thickness of fish gelatin/rice flour composite films (0.024–0.027 mm) increased when compared with film made from fish gelatin alone (0.020 mm). They also reported that the RF film-forming solution (FFS) had a high water-holding capacity, which led to a denser and thicker film when the RF-FFS was dry [12]. De Paola et al. [19] reported that the starch-based films blended with CMC at different levels showed the same thickness values (0.35–0.36 mm). The difference in the thickness of the obtained composite biopolymer films was generally affected by the nature of the component and the composition of the FFS.

### 3.2. Mechanical Properties

The chemical structure and nature of the starting film-forming material are associated with the films’ mechanical properties. Film prepared with CMCy exhibited a higher TS value (33.36 MPa), whereas the RF film showed a lower TS value (12.99 MPa) (Table 2). In the CMCy/RF composite films, the inclusion of RF at different levels affected the TS of the CMCy/RF composite biopolymer films. Significant decreases in the TS of the CMCy/RF composite films from 33.36 to 18.57 MPa were found when the amounts of RF increased (*p* < 0.05). This could be due to the presence of RF molecules dispersed, which caused the film matrix’s cohesion forces to weaken. In this way, a higher distributed RF content reduces cohesive forces and the film strength [12]. A decrease in the TS value was also observed in fish gelatin/RF blended films and CMC from durian rind/rice starch composite films when the amounts of RF and rice starch increased, respectively [6,12].

For EAB, the film made with CMCy had a higher EAB value (9.81%) than the RF film (3.95%) (Table 2). Among the CMCy/RF composite biopolymer films, the inclusion of RF at different levels affected the EAB of the CMCy/RF composite biopolymer films. Significant decreases in the EAB of the CMCy/RF composite films from 9.81 to 4.95% were observed when the levels of RF increased (*p* < 0.05). A similar result has been found in fish gelatin/rice flour blended films [12]. However, no significant difference was found in the CMCy50/RF50 composite film and the CMCy25/RF75 composite film. The distribution and density of intra- and intermolecular interactions, which are based on the orientation and arrangement of polymer chains in the film network, influence the mechanical properties of the resulting films [12]. According to these findings, adding RF at different levels changed the mechanical properties of the CMCy/RF composite biopolymer films.

### 3.3. Film Apperance and Color

**Figure 1:**
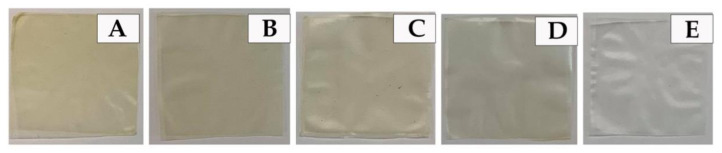
Appearance of (**A**) CMCy film, (**B**) CMCy75/RF25 composite film, (**C**) CMCy50/RF50 composite film, (**D**) CMCy25/RF75 composite film, and (**E**) RF film.

The appearance of the CMCy/RF composite biopolymer films produced in different proportions is presented in Figure 1. All film specimens had visually small particles and irregularities on the film’s surface. RF was compatible blended with CMCy at all formulations used. Visually, the CMCy film was light yellow, whereas the RF film was white and exhibited turbidity. The CMCy/RF composite films were slightly yellow to white. Increased RF levels tended to decrease the yellowness of the CMCy/RF composite films. This result was consistent with color results (Table 3). According to these findings, the appearance of the CMCy/RF composite films may affect consumer acceptance or the consumer’s decision to purchase when used as packaging films.

The color properties of film are a crucial factor that affects consumer acceptance in terms of packaging film application. The color parameters of the CMCy/RF composite biopolymer films produced in different proportions are presented in Table 3. The CMCy film had the highest b* value (yellowness) (7.06) and the lowest L* (lightness) (87.25) and a* (redness) (1.04) values. This indicated that the CMCy film was light yellow in color when compared with those of CMCy/RF composite biopolymer films. The yellow color of the CMCy film may be due to the carboxymethylation during the synthesized CMCy [1]. The RF film had the highest L* (90.14) and a* (2.75) values. The color of the CMCy/RF composite biopolymer films was influenced by the inclusion of RF in different amounts. The decreased b* value and the coincidental increase in L* and a* values were observed in the CMCy/RF composite biopolymer films compared to the CMCy film (*p* < 0.05). Suriyatem et al. [6] reported that blended films made from CMC from durian rind and rice starch had a yellowish color. Therefore, the films’ components, including the nature, concentration, and type of added biopolymer materials, had an effect on the color properties of the resulting composite films.

### 3.4. Film Transparency

**Figure 2:**
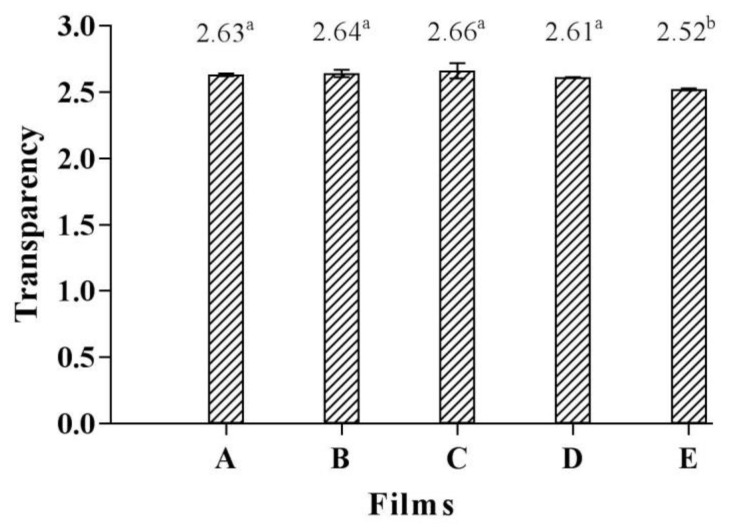
Transparency of (**A**) CMCy film, (**B**) CMCy75/RF25 composite film, (**C**) CMCy50/RF50 composite film, (**D**) CMCy25/RF75 composite film, and (**E**) RF film. Bars represent the standard deviation (*n* = 3). Different letters indicate the significant difference (*p* < 0.05).

Film transparency is an important property for many food applications, especially for the film used as see-through packaging. The transparency values of the CMCy film, CMCy/RF composite films produced in different proportions, and the RF film were 2.63, 2.61–2.66, and 2.52, respectively (Figure 2). The CMCy film and the CMCy/RF composite films showed a significant difference in transparency when compared with the RF film (*p* < 0.05). The transparency values of films made from CMCy alone and blended CMCy and RF were higher than those of the RF film. This indicated that the RF film was more transparent than those films containing CMCy. However, there was no significant difference in transparency between films made from CMCy blended with RF in different amounts (*p* > 0.05). Thus, the inclusion of RF did not affect the transparency of the CMCy/RF composite biopolymer films.

### 3.5. Water Vapor Permeability (WVP)

**Figure 3:**
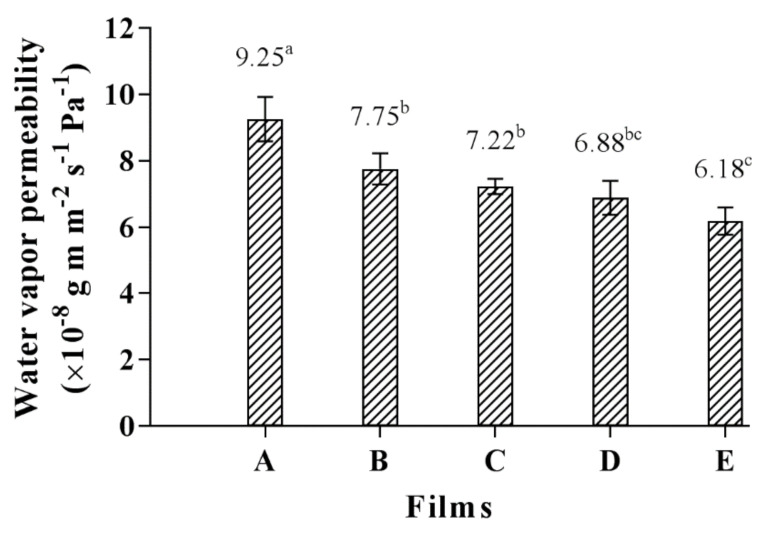
Water vapor permeability of (**A**) CMCy film, (**B**) CMCy75/RF25 composite film, (**C**) CMCy50/RF50 composite film, (**D**) CMCy25/RF75 composite film, and (**E**) RF film. Bars represent the standard deviation (*n* = 3). Different letters indicate the significant difference (*p* < 0.05).

The ability of composite biopolymer films to improve the storage or shelf life of various food products is ultimately measured by their water vapor barrier properties. The CMCy film had the highest WVP value (9.25 × 10^−8^ g m m^−2^ s^−1^ Pa^−1^) but the RF film showed the lowest WVP value (6.18 × 10^−8^ g m m^−2^ s^−1^ Pa^−1^); whereas the WVP values of the CMCy/RF composite films produced in different proportions were in the range of 7.75 and 6.88 × 10^−8^ g m m^−2^ s^−1^ Pa^−1^ (Figure 3). This indicated that the WVP value of the CMCy film decreased when the RF was added. This is because of the formation of intermolecular interactions between the carboxyl groups of CMCy and the hydroxyl groups of RF through hydrogen bonding. The bonding may decrease the number of available polar groups of the blended polymers, which may reflect the WVP of the resulting composite films. A similar result has been found in the composite films made from CMC from durian rind with rice starch [6]. However, no significant difference in WVP values was observed among the CMCy/RF composite films (*p* > 0.05). There are more water-binding and moisture-absorbing groups in the CMCy film, which means that it has a higher WVP than other films. Suriyatem et al. [6] also reported that film made with CMC from durian rind could absorb more water molecules than rice starch film due to it containing COO- groups. They also reported that the combinations of CMC from durian rind with rice starch did not affect the WVP of the composite films. The relative polarity of the polymer used, as well as the structure inside the film matrix, have a significant impact on WVP. Thus, the inclusion of RF could enhance the WVP of the CMCy film.

### 3.6. Film Solubility

**Figure 4:**
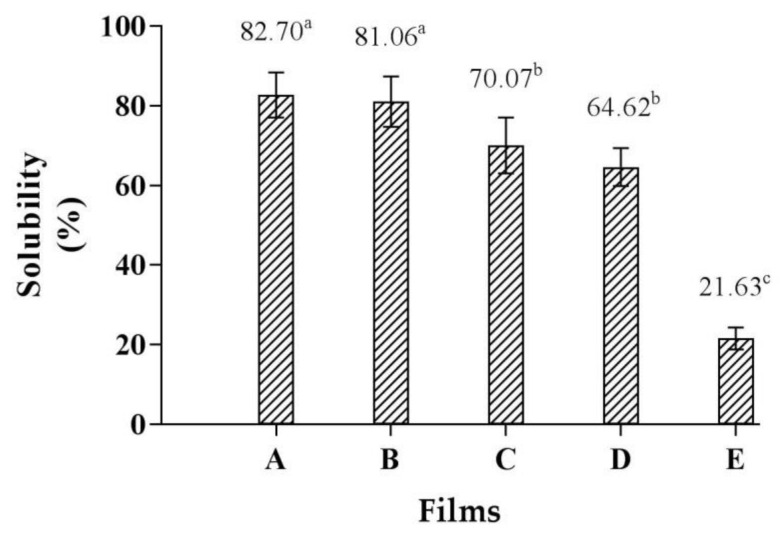
Film solubility of (**A**) CMCy film, (**B**) CMCy75/RF25 composite film, (**C**) CMCy50/RF50 composite film, (**D**) CMCy25/RF75 composite film, and (**E**) RF film. Bars represent the standard deviation (*n* = 3). Different letters indicate the significant difference (*p* < 0.05).

**Figure 5:**
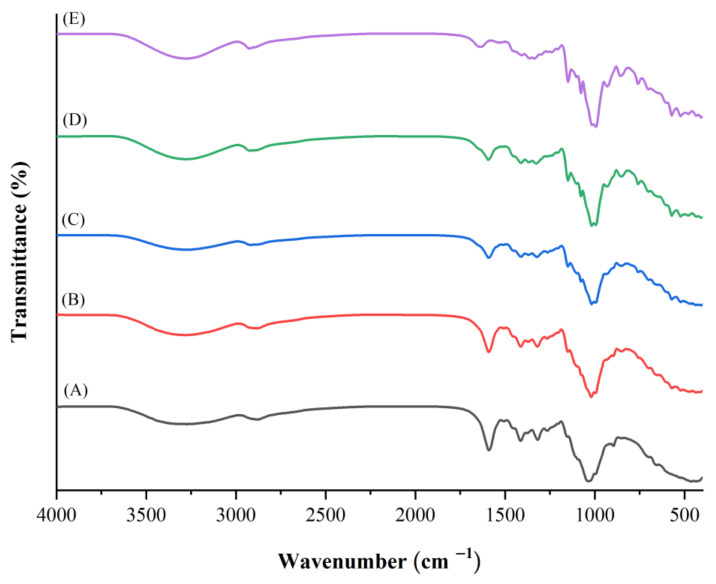
FTIR spectra of (**A**) CMCy film, (**B**) CMCy75/RF25 composite film, (**C**) CMCy50/RF50 composite film, (**D**) CMCy25/RF75 composite film, and (**E**) RF film.

The film solubility of the CMCy/RF composite biopolymer films produced in different proportions is presented in Figure 4. The CMCy film showed the highest film solubility (82.70%), whereas the RF film had the lowest film solubility (21.63%). The higher solubility of CMCy film was associated with its hydrophilic -CH_2_COONa groups, which interact with water molecules [26]. The film solubility of the CMCy film was significantly reduced from 82.70% to 64.62% when the levels of RF increased (0 to 75%) (*p* < 0.05). However, no significant difference was noted in the CMCy film and CMCy75/RF25 film, as well as the CMCy50/RF50 film and CMCy75/RF25 film (*p* > 0.05). The decreased solubility of CMCy/RF composite biopolymer films may be due to the interactions between the hydroxyl group of RF and the carboxyl group of CMC. These intermolecular interactions could improve the water resistance of CMCy/RF composite films. This result is in agreement with the structure analyzed by FTIR analysis (Figure 5). According to Tongdeesoontorn et al. [22], the CMC carboxyl groups and the CMC hydroxyl groups can form strong ester bonds and hydrogen bonds with the starch hydroxyl groups. As a result, the contacts between molecules are increased, the cohesiveness of the biopolymer matrix is improved, and the solubility of films is reduced. However, the level of intermolecular interactions is based on the CMC and starch contents [22]. This result was in accordance with Soo and Sarbon [13], who observed a reduction in the solubility (93.66–83.91%) of gelatin films when the content of rice flour increased from 0 to 25%. Ahmad et al. [12] also found that the film solubility (94.46–57.96%) of fish gelatin films decreased when increasing the amount of rice flour. During storage, transportation, and retailing, a decreased film solubility is needed, and a higher film solubility will be useful when coating food products with biopolymer-based films is required for cooking or to make them easily degradable in the natural environment. According to these findings, the addition of RF could reduce the solubility of CMCy/RF composite films.

### 3.7. FTIR Spectra Analysis

The FTIR spectrum of the CMCy film displayed major bands at 3274.17, 2874.50, 1589.37, and 1413.49 cm^−1^, referring to the –OH groups stretching, the C–H stretching vibration, antisymmetric stretching vibration of COO– groups, and symmetric stretching vibration of COO– groups, respectively [27]. The bands at 996.09 and 895.82 cm^−1^ are assigned to the –CH ring and C–C stretching vibrations, respectively [17] (Figure 5A).

In the FTIR spectrum of the RF film, the bands at 3278.59 and 2923.57 cm^−1^ were attributed to the –OH group stretching and the C–H stretching vibration of CH_2_ of starch, respectively [28]. Peaks at 1636.63 and 1148.80 cm^−1^ corresponded to water O–H bending and C–O–C antisymmetric bridge stretching, respectively [22] (Figure 5E).

For the CMCy/RF composite biopolymer films, the peak at 1589.37 cm^−1^ (CMCy film) was slightly shifted to 1590.43 cm^−1^ for the CMCy75/RF25 composite film, to 1590.66 cm^−1^ for the CMCy50/RF50 composite film, and to 1592.06 cm^−1^ for the CMCy25/RF75 composite film (Figure 5B–D). This suggested that antisymmetric and symmetric vibrations of C=O and C–O bonds were increased, most likely as a result of RF disrupting intermolecular hydrogen bonds between carboxylic groups [22].

The peak at 3274.17 cm^−1^ (CMCy film) referred to O–H stretching and intermolecular/intramolecular hydrogen bonding [22]. It was slightly shifted to 3276.41 cm^−1^ for the CMCy75/RF25 composite film, to 3275.30 cm^−1^ for the CMCy50/RF50 composite film, and to 3278.43 cm^−1^ for the CMCy25/RF75 composite film (Figure 5B–D). In the CMCy/RF composite biopolymer films, the peaks moved to higher wavenumbers because of the different conformations of the molecules caused by adding RF. This was associated with the reduction in the mechanical properties (both TS and EAB) in RF blended films.

The CMCy/RF composite biopolymer films showed a high wavenumber at a peak of 2885.66–2919.82 cm^−1^ (Figure 5B–D) compared to the CMCy film (2874.50 cm^−1^) (Figure 5A), suggesting that the interaction of CMCy and RF in the film matrix had occurred. In the CMCy/RF composite biopolymer films, the shift to a higher wavenumber indicated conformational modifications of functional groups or an interaction with bigger MW groups [12].

According to these findings, these peaks were minimally shifted to different higher wavenumbers when the levels of RF increased, and were mostly related to the properties of films changing. Similar results have been found in CMC from durian rind/rice starch composite films [6], fish gelatin/rice flour blended films [12], CMC/alginate/chitosan [15], rice starch/CMC blended films [17], and also CMC/cassava starch blended films [22]. The shift in the wavenumbers of the films demonstrates the interactions between the carboxyl groups of CMCy and the hydroxyl groups of RF. This means that hydrogen bonds acting on the COO– for the CMCy/RF composite biopolymer films may be weaker than those of the CMCy film. Therefore, FTIR spectra confirmed the changes and interactions that occurred in the CMCy/RF composite biopolymer films.

### 3.8. Film Morphology

**Figure 6:**
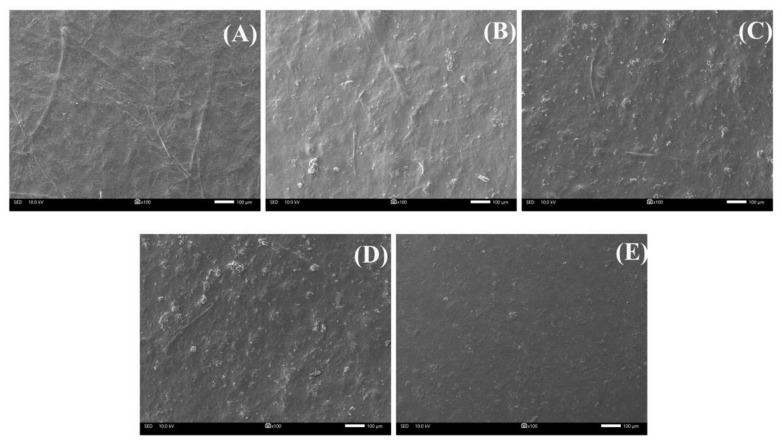
SEM micrograph surface of (**A**) CMCy film, (**B**) CMCy75/RF25 composite film, (**C**) CMCy50/RF50 composite film, (**D**) CMCy25/RF75 composite film, and (**E**) RF film.

Scanning electron microscopic (SEM) micrographs of the surface of the CMCy/RF composite biopolymer films produced in different proportions are presented in Figure 6. The CMCy film had a coarse, wrinkled, and irregular surface, and it contained a CMCy filament structure. This may be due to the fact that the water solubility of CMCy was 74.98% [1], which caused the presence of insoluble fibrous character during the film forming process. The RF film showed residue of starch granules, roughness, and an irregular surface. According to Dias et al. [11] and Vargas et al. [29], irregularities on the surface of RF-based films are caused by the presence of varying macromolecules in the polymer film matrix, such as starch, proteins, lipids, and fiber, as well as interactions between these components. Among the CMCy/RF composite biopolymer films, the inclusion of RF in the CMCy film resulted in a coarse, protruding, wrinkled, and irregular surface, regardless of the ratios used. In addition, the filament structure of CMCy and starch granule residues were found on the surface of films made with blended CMCy and RF. This was the same as in films made with CMCy alone or RF alone. This might reflect the involvement of CMCy and RF molecules through strong bonding, which might have occurred due to the carboxyl groups of CMCy and the hydroxyl groups of RF present in the polymer. This interaction supports other findings, such as a low TS, EAB, WVP, and solubility, which demonstrate that these are effective combinations for composite bioloymer film formation. The difference in the microstructure of different biopolymer films was caused by the various polymer chain arrangements during the formation of films [12]. Rai et al. [14] reported that the inclusion of RF to pectin-based edible films showed a compact, irregular structure with small starch granules (some insoluble fractions). However, films made from CMC from durian rind blended with rice starch showed a homogenous and smooth surface [6]. Soo and Sarbon [13] reported that the addition of RF at a low level (5%) to chicken skin gelatin film showed a flat, compact, and homogenous surface, whereas a slightly protruded and irregular surface was observed when a high level (25%) of RF was added. Thus, the film microstructure was governed by molecular organization in the film matrix, which was based on component types, component interactions in the film matrix, and component ratios used to make composite biopolymer films [12].

## 4. Conclusions

The mechanical and physicochemical properties of the CMCy/RF composite biopolymer films were significantly influenced by the inclusion of RF. Increased levels of RF in the CMCy/RF composite films tended to reduce film strength and flexibility. Water vapor permeability and film solubility values were lowered in films made with blended CMCy/RF. However, the addition of RF did not influence the thickness and transparency of the CMCy/RF composite films. The CMCy/RF composite films had a yellow to white color, and their yellowness was slightly decreased as the RF proportions increased. FTIR spectra showed that some interactions between CMCy and RF blended films had occurred. On SEM micrographs, all film samples had a coarse, protruding, wrinkled, and irregular surface. According to these findings, blending CMCy and RF at a proportion of the CMCy50/RF50 composite biopolymer film was found to be the best formulation, as it resulted in moderate mechanical and adequate physicochemical properties. In addition, the CMCy50/RF50 composite biopolymer film has the potential to be used as a composite biopolymer film material.

## Figures and Tables

**Table 1:** The thickness of the CMCy/RF composite biopolymer films produced in different proportions.

Films	Thickness (mm)
CMCy100	0.056 ± 0.0012 ^a^
CMCy75/RF25	0.057 ± 0.0019 ^a^
CMCy50/RF50	0.056 ± 0.0011 ^a^
CMCy25/RF75	0.056 ± 0.0012 ^a^
RF100	0.058 ± 0.0012 ^b^

Value is given as mean ± SD from *n* = 10. Different superscripts in column are significantly different (*p* < 0.05). Numbers denote the ratios of CMCy/RF.

**Table 2:** Tensile strength (TS) and elongation at break (EAB) of the CMCy/RF composite biopolymer films produced in different proportions.

Films	TS (MPa)	EAB (%)
CMCy100	33.36 ± 1.59 ^a^	9.81 ± 0.82 ^a^
CMCy75/RF25	24.98 ± 1.16 ^b^	7.90 ± 1.07 ^b^
CMCy50/RF50	23.25 ± 1.98 ^c^	5.38 ± 0.71 ^c^
CMCy25/RF75	18.57 ± 0.77 ^d^	4.95 ± 0.53 ^c^
RF100	12.99 ± 0.79 ^e^	3.95 ± 0.59 ^d^

Values are given as mean ± SD from *n* = 7. Different superscripts in each column are significantly different (*p* < 0.05). Numbers denote the ratios of CMCy/RF.

**Table 3:** Color parameters of the CMCy/RF composite biopolymer films produced in different proportions.

Films	L*	a*	b*
CMCy100	87.25 ± 0.51 ^c^	1.04 ± 0.08 ^e^	7.06 ± 0.52 ^a^
CMCy75/RF25	88.38 ± 0.16 ^b^	1.25 ± 0.11 ^d^	4.08 ± 0.57 ^b^
CMCy50/RF50	88.38 ± 0.72 ^b^	1.49 ± 0.10 ^c^	2.54 ± 1.10 ^c^
CMCy25/RF75	89.57 ± 0.29 ^a^	1.72 ± 0.07 ^b^	0.04 ± 0.40 ^d^
RF100	90.14 ± 0.35 ^a^	2.75 ± 0.04 ^a^	−5.00 ± 0.08 ^e^

Values are given as mean ± SD from *n* = 3. Different superscripts in each column are significantly different (*p* < 0.05). Numbers denote the ratios of CMCy/RF.

## Data Availability

The data presented in this study are available on request from the corresponding author.
